# Knowledge, attitude and behaviors towards patients with mental illness: Results from a national Lebanese study

**DOI:** 10.1371/journal.pone.0222172

**Published:** 2019-09-16

**Authors:** Carla Abi Doumit, Chadia Haddad, Hala Sacre, Pascale Salameh, Marwan Akel, Sahar Obeid, Maria Akiki, Elie Mattar, Najla Hilal, Souheil Hallit, Michel Soufia

**Affiliations:** 1 Faculty of Medicine and Medical Sciences, Holy Spirit University of Kaslik (USEK), Jounieh, Lebanon; 2 Psychiatric Hospital of the Cross, Jal Eddib, Lebanon; 3 Drug Information Center, Order of Pharmacists of Lebanon, Beirut, Lebanon; 4 INSPECT-LB: Institut National de Sante Publique, Epidemiologie Clinique et Toxicologie, Beirut, Lebanon; 5 Faculty of Medicine, Lebanese University, Beirut, Lebanon; 6 Faculty of Pharmacy, Lebanese University, Beirut, Lebanon; 7 School of Pharmacy, Lebanese International University, Beirut, Lebanon; 8 Faculty of Philosophy and Human Sciences, Holy Spirit University of Kaslik (USEK), Jounieh, Lebanon; 9 Faculty of Pedagogy, Lebanese University, Beirut, Lebanon; Institute of Mental Health, SINGAPORE

## Abstract

**Objectives:**

Patients with mental health disorders often have to endure the burdens of the condition itself and the stigma that follows. Since no study has been conducted in Lebanon on this topic, our objective was to assess the knowledge, attitude and behaviors towards public stigma of mental health diseases, among a sample of the Lebanese population.

**Methods:**

A cross-sectional study, conducted between November 2017 and May 2018, enrolled 2289 participants. The Mental Health Knowledge Schedule (MAKS), the Community Attitudes toward Mental Illness (CAMI) and the Reported and Intended Behavior Scale (RIBS) were used to assess knowledge, attitude and behaviors toward mental illness respectively. The 25th, 50th and 75th percentile of the MAKS, CAMI and RIBS scales scores were considered as cutoff points for low, medium and high scores respectively.

**Results:**

A high knowledge score was found in 33.0% of the participants, whereas a high attitude score and a higher behavior score were found in 32.2% and 26.9% of the participants respectively. Living in North Lebanon (Beta = 1.331) and being familiar with a non-close person with mental illness (Beta = 0.811) were associated with higher knowledge of mental illness (higher MAKS score), whereas living in Bekaa (Beta = -8.693) and being 70 years old and above (Beta = -5.060) were associated with lower knowledge toward mental illness (lower MAKS score). Higher knowledge of mental illness (higher MAKS score) (Beta = 0.670), having a high level of education (university (Beta = 8.785), secondary (Beta = 6.084) and technical (Beta = 5.677)) were associated with less stigmatizing attitudes (higher CAMI scale). Being familiar with close people with mental illness (Beta = 0.577), less stigmatizing attitudes (higher CAMI scale) (Beta = 0.077) and higher knowledge of mental illness (higher MAKS score) (Beta = 0.115) were associated with higher favorable behaviors (higher RIBS score), whereas knowing a non-close person who have a mental illness (Beta = -0.720) was associated with lower favorable behaviors (lower RIBS score).

**Conclusion:**

A mass media awareness campaigns that could transmit health messages to a wide public audience in the country to fight stigma toward mental illness, seems warranted.

## Introduction

Patients with mental health disorders do not only have to endure the burden of having the condition but also the stigma that results from it, and that is classified into: public stigma, institutional stigma, and self-stigma. In this paper, we will address public stigma and refer to it as stigma. Stigma is defined as “a set of negative attitudes and beliefs that motivate individuals to fear, reject, avoid, and discriminate against people with mental illness” [1, 2]. It results in reduced autonomy and self-efficacy [3]. Stigma is mainly manifested in three ways: stereotype, prejudice and discrimination [2]. Many nations have taken steps forward to fight this social phenomenon. An example would be the United Kingdom and its “Time to Change” campaign [4]. Similar campaigns have been launched in New Zealand (Like Minds Like Mine) [5], Denmark (One of Us) [6], and Canada (Opening Minds) [7]. These anti-stigma initiatives targeted not only the general population but also specific groups through social media.

Studies have shown that low rates of seeking psychiatric help are mainly due to poor knowledge of mental health disorders (MHD) [8], that includes information about mental disorders, symptoms, and psychiatric treatments [9]. Many studies have also shown that more knowledge leads to fewer stigma [10, 11]. Moreover, attitudes range from acceptance [12] and tolerance [13] to negativity and fear [14]. When a positive attitude is portrayed, a supportive and open-minded behavior follows such as hiring a person suffering from MHD. Conversely, when attitudes are negative, it results in avoidance, social exclusion and discrimination [15].

Previous findings have shown that being in contact with a person having MHD influences behaviors, emotions and attitudes [16, 17]. In some cases, having experience with mental illnesses generated a positive and an understanding attitude, while in other cases, there has been rejection and negativity [18]. Interpersonal contact with patients with mental illness could develop understanding attitudes, change the beliefs, and reduce misconceptions toward these patients [19]. However, people may hold some negative views about the dangerousness of people with MHD and would prefer to keep a social distance despite their regular contact with them [20]. A study conducted by Angermeyer et al. showed that a large part of the public cannot recognize a specific mental disorder and the majority of the public consider people with mental disorders to be in need of help [21]. However, a substantial part perceives them as dangerous and unpredictable and reacts with fear [21]. In addition, several studies have shown that the general public perceives individuals with mental illness to be dangerous to themselves and others [22–25]. A worldwide study conducted in 229 countries showed that in developed countries such as the USA and Canada, only 7% to 8% of respondents had stigma towards patients with MHD, compared to 15% or 16% in developing countries [26], where people stigmatize, fear and distance themselves from patients with MHD; they also show prejudices and stereotypes as they think that patients with mental illnesses tend to be more violent.

In most Arab countries stigma toward mental illness is still prevalent and people with MHD still experience the disadvantages of poverty and illness stigma [27]. Arab countries, have shared set of values, traditions and beliefs that are different from those of the western countries [28]. In Arab countries, patients with MHD have a negative attitude toward mental health services and tend to avoid the use these services; they express their psychological problems in the form of physical symptoms [27]. In Arab countries, symptoms of psychiatric disorders are associated to religious beliefs [29]. Most of mentally ill patients of the Arab world are first examined by the religious or spiritual healer whose task is to free the patient from the “evil” [29]. A large number of Arabs-speaking persons in Australia believes that mental illness is an experience of God because it is the result of sin or wrongdoing [30]. Consequently, socio-cultural, religious and political aspects of the Arab world have an impact on psychiatric care. A study conducted in Egypt among 208 participants recruited through their places of work showed that the majority of respondents (70.2%) do not accept a person with MHD as a teacher for their children, 53.7% do not accept him as a family member, 32.7% do not accept him as a friend and 25.1% do not accept him as a neighbor. In this study, patients with psychiatric disorders are stigmatized and often face social rejection [31]. Another study conducted in the United Arab Emirates among parents of children with MHD showed that the majority of parents (62%) often do not seek help from mental health specialists [32].

Lebanon is an Arab country located in the Eastern Mediterranean region, with high religiosity among its eighteen various religious communities. A Lebanese study of a sample of 203 undergraduates revealed that stigma differs considerably according to various cultural misconceptions, for example 158 (77.8%) they think that evil eye might cause mental illness and 95 (46.8%) think that mental illness is a punishment from God [33]. The Arab World has its distinctive sociocultural beliefs about mental illness; Arabs profoundly believe in the reality of paranormal entities such as evil eye, Jinn (the devil), Sehr (Black Magic). They associate the symptoms of psychiatric disorders to the workings of these paranormal entities [33]. In 2010, a report of the WHO (World Health Organization) showed the existence of 3 mental hospitals and 5 community-based psychiatric inpatient units in Lebanon [34]. A total of 42 psychiatric beds are available per 10,000 inhabitants, and 2 psychiatrists per 100,000 inhabitants. Patients admitted to a hospital for mental illnesses mainly belong to the following two diagnostic groups: schizophrenia, schizotypal and delusional disorders (47%) and mood disorders (12%) [35]. A study done by Karam et al. among a representative sample of the Lebanese population (n = 2857) had showed that 25.8% of the sample met at least one of the criteria of the DSM-IV (Diagnostic and Statistical Manual of Mental Disorders—fourth edition) at some point in their lives [36]. In Lebanon, stigma is still widespread with regard to mental illness, and people diagnosed with MHD still hide their disease: instead of seeking medical help, they refuse it because of cultural stigma [33].

To our knowledge two studies had been done in Lebanon that evaluated knowledge, attitudes and beliefs regarding mental illness [33, 37]. The first one was done among undergraduate people and the second among Catholic clerics [33, 37]. However, no studies had been done among the general population that evaluate knowledge, attitude and behaviors towards the stigmatization of MHD. Therefore, the objective of this study is to assess these parameters among a sample of the Lebanese population.

## Methods

### Ethical approval

In accordance with the hospital’s Regulatory Research Protocol, the Ethics and Research Committee of the Psychiatric Hospital of the Cross, Jal Eddib, Lebanon approved this study protocol (HPC-023-2018) based on the fact that the autonomy and confidentiality of participants were respected and that it was an observational study with no prejudice to them. The purpose and requirements of the study were communicated to each participant. Consent was obtained in the form of written approval of the ethical consent form.

### Study design and sample

This cross-sectional study was conducted between November 2017 and May 2018; it enrolled 2289 community dwelling participants using a proportionate random sample from all Lebanese governorates (Beirut, Mount Lebanon, North, South and Bekaa). Each governorate is divided into Caza (stratum). In the first stage of the random sampling technique, two villages were randomly selected from each Caza, based on the list of villages provided by the Central Agency of Statistics in Lebanon. In the second stage, in each selected village, the questionnaire was distributed randomly to the households, based on random sampling technique to select the included house. All persons living inside the selected house were invited to participate, if eligible. After eligibility criteria were determined, subjects were assigned identification numbers and randomized according to an online software, Research Randomizer (www.randomizer.org). The stratified randomization method was used since it allows to control and balance the influence of covariates. Prior to participation, individual subjects were briefed on the study objectives and methodology, and were assured of the anonymity of their participation. Individuals agreeing to participate in the study were then asked to read through and sign off a written informed consent form. Those who accepted to participate in the study were invited to fill out the questionnaire via a face-to-face interview. All participants above 18 years of age were eligible to participate. Excluded were the persons with self-reported psychiatric problems or those who refused to participate. No resources/helpline brochures were given to participants.

### Questionnaire

The questionnaire included 102 questions and was in Arabic (the native language in Lebanon). The first part covered socio-economic and demographic characteristics, including age, gender, marital status and the level of education. The level of education was divided into four categories: primary, secondary, university and technical education–the technical education system in Lebanon is defined as a curriculum (combination of theoretical and practical studies) that prepares skilled technicians [38]. The household crowding index was calculated by dividing the number of persons living in the house by the number of rooms in the house excluding the bathroom(s) and the kitchen. The higher the score the more the house is crowded. Overcrowded households are often households with few economic resources [39].

The second part, included three scales: the Mental Health Knowledge Schedule (MAKS), the Community Attitudes toward Mental Illness (CAMI), and the Reported and Intended Behaviour Scale (RIBS). All three scales were not validated in Arabic. Therefore, all scales were translated from English to Arabic through an initial translation and back translation process. The English version was translated into Arabic by a mental health specialist, then back-translated into English by another specialist. Upon completion of this process, the translators compared the English versions of all the scales to determine whether the variables had the same meaning. An expert committee formed by healthcare professionals and a language professional verified the Arabic translated version. The expert committee aimed at discerning discrepancies and to solve any inconsistencies between the two versions. The process of forward-back translation was repeated until all ambiguities disappeared.

#### The Mental Health Knowledge Schedule (MAKS)

This twelve-item scale comprises domains of relevant evidence-based knowledge in relation to stigma toward mental illness. Items are coded on an ordinal scale (1–5). Items which the respondent strongly agrees with score 5 points; 1 point reflects a response to which the respondent strongly disagrees. The total score is calculated by adding the points obtained for each of the 12 items. Higher total scores correspond to greater knowledge [40]. The Cronbach’s alpha for the MAKS scale was 0.749.

#### The Community Attitudes toward Mental Illness (CAMI)

The forty-item CAMI scale was developed by two researchers at a Canadian university [41] and was used in this study to measure public stigma attitudes towards mental illness. All items are rated according to a five-point Likert scale (1 = strongly agree to 5 = strongly disagree). Negatively stated items were reversely recoded for analysis. The scale has four subscales, each with 10 items: Authoritarianism (AU), Benevolence (BE), Social Restrictiveness (SR), and Community Mental Health Ideology (CMHI). AU is a “view of the mentally ill person as someone who is inferior and requires supervision and coercion.” BE corresponds to “a humanistic and sympathetic view of mentally ill persons”; SR means “the belief that mentally ill patients are a threat to society and should be avoided.” Community Mental Health Ideology (CMHI) is “the acceptance of mental health services and the integration of mentally ill patients in the community” [41]. Higher AU scores, lower BE scores, lower SR scores and higher CMHI scores would indicate higher stigma. Overall stigma against patients with mental illness was computed by summing up the subscales. Higher scores indicated less stigma attitudes against patients with mental illness. The Cronbach’s alpha for the total scale and subscales were as follows: CAMI (0.876), AU (0.555), BE (0.637), SR (0.690) and CMHI (0.804).

#### The Reported and Intended Behavior Scale (RIBS)

The RIBS (eight-item scale) comes in two groups of four items each. The first group focuses on behavior reported in past or present experiences regarding the following areas: live with, work with, live nearby, or have a relationship with a person with a mental health problem. The second group focuses on future intentions to establish contact with people with a mental health problem in the same areas as described above. Each item is coded on an ordinal scale (1 = strongly disagreed to 5 = strongly agreed). “Do not know” is coded as neutral. High values correspond to more favorable expected behaviors [42]. The Cronbach’s alpha for the CAMI scale was 0.766.

### Data collection

Data collection was performed by study-independent clinical psychologists who had received a thorough training prior to data collection, and whose role was to evaluate participants’ level of mental illness to exclude those with psychiatric problems. No resources/helpline brochures were given to participants. The questionnaire was completed within 45 minutes approximately. During the data collection process, the anonymity of the participants was assured. Individual participants had the right to accept or refuse participation in the study, with no financial compensation provided in exchange for individual participation.

### Statistical analysis

Data analysis was conducted using SPSS software version 23. The independent-sample t-test was used when comparing two means, whereas the ANOVA test was used to compare 3 means or more. The Pearson’s correlation coefficient was used between 2 quantitative variables. Since better knowledge would lead to better attitudes, which would lead to better behaviors, three hierarchical stepwise linear regressions were conducted; in the first one, we took the MAKS score (knowledge score) as the dependent variable and sociodemographic variables as independent variables. In the second one, we took the CAMI score (attitude score) as the dependent variable, with the sociodemographic variables and the knowledge score as independent variables. Finally, in the third regression, we considered the RIBS score (behaviors score) as the dependent variable and the sociodemographic variables, knowledge and attitude scores as independent variables. All variables that showed a p<0.1 in the bivariate analysis were taken as independent variables in the regression model in order to eliminate the potential confounding factors. Moreover, Cronbach’s alpha was recorded for reliability analysis for all the scales. A p-value less than 0.05 was considered significant.

## Results

Overall, 2289 persons out of 3000 completed the interviews and 711 refused to participate; thus, the response rate was 76.3%. More than half of the participants were females (53.0%), unemployed (60.9%), between 18 and 24 years old (61.0%), and university graduates (62.4%) (Table 1).

**Table 1:** Sociodemographic characteristics of the participants.

		Frequency (%)
Age	18–24 years	1342 (61.0%)
	30–49 years	580 (26.4%)
	50–69 years	255 (11.6%)
	>70 years	22 (1.0%)
Sex	Male	1032 (47.0%)
	Female	1163 (53.0%)
Education level	Primary	105 (4.7%)
	Secondary	541 (24.2%)
	University	1393 (62.4%)
	Technical education	194 (8.7%)
Employment status	Employed	862 (39.1%)
	Unemployed	1347 (60.9%)
Region	Beirut	441 (20.2%)
	Mont Lebanon	979 (44.8%)
	North	278 (12.7%)
	South	348 (15.9%)
	Bekaa	141 (6.4%)
		**Mean ± SD**
The household crowding index		0.64 ± 0.35

Based on the total scores of CAMI, MAKS and RIBS scales, 25^th^, 50^th^ and 75^th^ percentile were considered as cut off points for low, medium and high score. A higher score of public stigma toward mental illness was found in 67.8% of the participants. The higher score of knowledge toward mental illness was 61.9% and 66.6% had more favorable behaviors. The mean scores for all the scales and subscales were as follows: CAMI (136.84 ± 17.55), AU (32.74 ± 5.05), BE (36.75 ± 5.25), SR (34.34 ± 5.50), CMHI (33.19 ± 6.25), MAKS (39.00 ± 6.84) and RIBS (15.58 ± 4.10).

### Bivariate analysis

The bivariate analysis of factors associated with the total CAMI scale score showed a significantly higher mean CAMI score (less stigma) in females compared to males (138.62 vs. 135.58, p<0.001), in those with a university level of education compared to a primary one (138.74 vs. 127.96, p<0.001), and in those aged between 18–24 years compared to those above 70 years (138.25 vs. 126.77, p<0.001). Also, people who live in Beirut had a significantly higher mean CAMI scale than people who live in Bekaa (137.31 vs. 131.34, p<0.001). A higher CAMI score was significantly associated with higher MAKS (r = 0.689) and RIBS scores (r = 0.778).

The bivariate analysis taking the MAKS score as the dependent variable, showed a significantly higher mean MAKS score in females compared to males (39.41 vs. 38.62, p = 0.007), in those with a university level of education compared to a primary one (39.30 vs. 37.34, p = 0.001), in those aged between 18–24 years compared to those above 70 years (39.51 vs. 34.45, p = 0.005) and in those living in Mount Lebanon compared to those living in Bekaa (39.66 vs. 30.65, p<0.001). A higher MAKS score was significantly associated with a higher household crowding index (r = 0.055), CAMI scale (r = 0.689), RIBS scale (r = 0.709), the number of family/friends (close people) (r = 0.077) and non-close people (r = 0.082) with mental illness the person knows.

The bivariate analysis taking the RIBS score as the dependent variable showed a significantly higher mean RIBS score in people living in Beirut compared to those living in Bekaa (16.40 vs. 13.81, p<0.001). A higher RIBS score was also significantly associated with the number of family/friends (close people) (r = 0.143) and non-close people (r = 0.087) with mental illness the person knows (Tables 2 and 3).

**Table 2:** Bivariate analysis of sociodemographic factors associated with each subscale of the CAMI score.

Variable	Total CAMI score	AU	BE	SR	CMHI	MAKS	RIBS
**Age categories**							
18–29 years	138.25 ± 16.91	33.17 ± 4.93	37.06 ± 5.18	34.89 ± 5.51	33.45 ± 6.06	38.96 ± 6.99	15.65 ± 3.91
30–49 years	135.58 ± 18.54	32.28 ± 5.47	36.54 ± 5.36	33.80 ± 5.53	33.03 ± 6.50	39.51 ± 6.52	15.52 ± 4.38
50–69 years	134.65 ± 17.75	32.03 ± 4.58	36.42 ± 5.01	33.25 ± 5.09	32.78 ± 6.62	38.32 ± 7.01	15.35 ± 4.22
70 years and above	126.77 ± 19.50	29.95 ± 6.27	36.84 ± 5.21	30.95 ± 6.78	29.50 ± 6.58	34.45 ± 7.10	14.09 ± 4.70
p-value	**<0.001**	**<0.001**	0.095	**<0.001**	**0.01**	**0.005**	0.438
**Gender**							
Male	135.58 ± 17.93	32.33 ± 5.10	36.50 ± 5.41	33.74 ± 5.54	33.12 ± 6.32	38.62 ± 7.08	15.43 ±3.97
Female	138.62 ± 16.85	33.29 ± 4.93	37.19 ± 4.94	35.05 ± 5.41	33.39 ± 6.17	39.41 ± 6.51	15.67 ±4.12
p-value	**<0.001**	**<0.001**	**0.002**	**<0.001**	0.307	**0.007**	0.18
**District**							
Beirut	137.31 ± 16.65	32.58 ± 4.88	36.71 ± 5.36	34.63 ± 5.66	33.77 ± 5.44	39.54 ± 6.01	16.40 ± 4.26
Mount Lebanon	137.11 ± 16.82	32.86 ± 5.22	36.88 ± 4.86	34.44 ± 5.46	33.05 ± 6.25	39.66 ± 6.23	15.55 ± 3.97
North	137.08 ± 19.66	32.64 ± 5.24	37.10 ± 5.10	34.11 ± 5.95	33.26 ± 7.63	39.90 ± 6.06	15.30 ± 4.69
South	135.43 ± 17.70	32.62 ± 4.48	36.07 ± 5.68	34.29 ± 5.35	32.88 ± 5.94	39.43 ± 5.79	15.58 ± 3.81
Bekaa	131.34 ± 11.36	31.39 ± 2.62	36.47 ± 4.58	31.80 ± 3.51	31.26 ± 4.63	30.65 ± 10.35	13.81 ± 3.08
p-value	**0.002**	**<0.001**	0.078	**<0.001**	**0.001**	**<0.001**	**<0.001**
**Education level**							
Primary	127.96 ± 19.72	30.07 ± 5.58	35.11 ± 5.56	31.57 ± 5.35	31.17 ± 7.38	37.34 ± 6.86	14.87 ± 5.12
Secondary	135.63 ± 16.84	32.51 ± 4.88	36.32 ± 5.07	33.72 ± 5.34	33.13 ± 5.98	39.10 ± 6.20	15.42 ± 4.25
University	138.74 ± 17.15	33.22 ± 4.88	37.26 ± 5.11	35.00 ± 5.40	33.57 ± 6.23	39.30 ± 6.91	15.74 ± 3.91
Technical	134.57 ± 16.60	32.31 ± 5.06	36.26 ± 5.20	33.35 ± 5.64	32.65 ± 5.72	38.00 ± 7.31	15.28 ± 4.11
p-value		**<0.001**	**<0.001**	**<0.001**	**0.001**	**0.001**	0.114
**Knowing someone with a mental illness**							
Yes	137.01 ± 16.91	32.43 ± 4.99	36.79 ± 5.03	34.46 ± 5.61	33.49 ± 6.17	39.94 ± 6.05	16.18 ± 4.33
No	137.88 ± 17.18	32.23 ± 5.07	37.19 ± 4.94	34.41 ± 5.31	33.13 ± 6.32	38.31 ± 7.31	14.97 ± 3.79
p-value	0.273	**0.001**	0.084	0.842	0.214	**<0.001**	**<0.001**

Authoritarianism (AU), Benevolence (BE), Social Restrictiveness (SR), and Community Mental Health Ideology (CMHI); MAKS: Mental Health Knowledge Schedule; RIBS: reported and Intended Behavior Scale. Lower scores on “Authoritarian” and “Social Restrictiveness” signify greater amounts of stigma, while lower on “Benevolence” signifies higher stigma and a higher score on “Community Mental Health Ideology” signifies higher acceptance of the mentally ill.

**Table 3:** Correlation between continuous variables and the CAMI subscales, MAKS and RIBS scores.

Variable	AU	BE	SR	CMHI	MAKS	RIBS
**AU**						
r	1	0.517	0.603	0.522	0.663	0.687
p-value	-	**<0.001**	**<0.001**	**<0.001**	**<0.001**	**<0.001**
**BE**						
r	0.517	1	0.528	0.475	0.669	0.673
p-value	**<0.001**	-	**<0.001**	**<0.001**	**<0.001**	**<0.001**
**SR**						
r	0.603	0.528	1	0.547	0.596	0.546
p-value	**<0.001**	**<0.001**	-	**<0.001**	**<0.001**	**<0.001**
**CMHI**						
r	0.522	0.475	0.547	1	0.609	0.594
p-value	**<0.001**	**<0.001**	**<0.001**	-	**<0.001**	**<0.001**
**MAKS**						
r	0.663	0.669	0.596	0.609	1	0.609
p-value	**<0.001**	**<0.001**	**<0.001**	**<0.001**	-	**<0.001**
**RIBS**						
r	0.687	0.673	0.546	0.594	0.609	1
p-value	**<0.001**	**<0.001**	**<0.001**	**<0.001**	**<0.001**	-
**Familiarity with close PWMI**						
r	-0.019	-0.053	-0.014	-0.006	0.041	0.143
p-value	0.359	**0.011**	0.5	0.764	0.054	**<0.001**
**Familiarity with non-close PWMI**						
r	-0.018	0.001	0.033	0.041	0.082	0.087
p-value	0.386	0.944	0.121	**0.049**	**<0.001**	**<0.001**
**House crowding index**						
r	-0.1	-0.071	-0.081	-0.027	-0.125	-0.028
p-value	**<0.001**	**0.001**	**<0.001**	0.206	**<0.001**	0.179

Authoritarianism (AU), Benevolence (BE), Social Restrictiveness (SR), and Community Mental Health Ideology (CMHI); MAKS: Mental Health Knowledge Schedule; RIBS: reported and Intended Behavior Scale, **PWMI: person with mental illness.** Higher scores on “Authoritarian” and “Social Restrictiveness” signify greater amounts of stigma, while higher scores on “Benevolence” signifies lower stigma and a higher score on “Community Mental Health Ideology” signifies higher acceptance of the mentally ill.

### Multivariable analysis

A first linear regression, taking the MAKS scale as the dependent variable, showed that living in North Lebanon (Beta = 1.331) and knowing non-close people with mental illness (Beta = 0.811) were associated with higher knowledge of mental illness (higher MAKS scores), whereas living in Bekaa (Beta = -8.693) and being 70 years old and above (Beta = -5.060) were associated with lower knowledge toward mental illness (lower MAKS scores).

A second linear regression, taking the CAMI scale as the dependent variable, showed that higher knowledge of mental illness (higher MAKS score) (Beta = 0.670) and having a high level of education (university (Beta = 8.785), secondary (Beta = 6.084) and technical (Beta = 5.677)) were associated with less stigmatizing attitudes (higher CAMI scores).

A third linear regression, taking the RIBS scale as the dependent variable, showed that knowing close people with mental illness (Beta = 0.577), less stigmatizing attitudes (higher CAMI scores) (Beta = 0.077) and higher knowledge of mental illness (higher MAKS scores) (Beta = 0.115) were associated with higher favorable behaviors (higher RIBS scores), whereas knowing a non-close person who have a mental illness (Beta = -0.720) was associated with lower favorable behaviors (lower RIBS score) (Table 4).

**Table 4:** Multivariable analysis.

**Model 1: Linear regression taking the MAKS scale (knowledge score) as the dependent variable.**					
	**Unstandardized Beta**	**Standardized Beta**	**p-value**	**Confidence interval**	
				**Lower Bound**	**Upper Bound**
Bekaa	-8.693	-0.314	<0.001	-9.894	-7.492
Familiarity with non-close people with mental illness (yes vs no*)	0.811	0.063	0.010	0.190	1.432
Age 70 years and above compared to 18–29 years*	-5.060	-0.069	0.002	-8.221	-1.900
North compared to Beirut*	1.331	0.063	0.004	0.418	2.243
Variables entered: Sex, Age, the household crowding index, Familiarity close people, Region, education level, familiarity non-close people who have mental illness.					
**Model 2: Linear regression taking the total CAMI scale (attitude score) as the dependent variable.**					
	**Unstandardized Beta**	**Standardized Beta**	**p-value**	**Confidence interval**	
				**Lower Bound**	**Upper Bound**
Knowledge toward mental illness score (MAKS scale)	0.670	0.266	<0.001	0.570	0
University education compared to primary*	8.785	0.253	<0.001	6.154	11.416
Secondary education compared to primary*	6.084	0.152	<0.001	3.264	8.904
Technical education compared to primary*	5.677	0.094	0.001	2.373	8.981
Variables entered: Sex, the household crowding index MAKS scale, Familiarity with close people who have mental illness, Age categories, Region, education level.					
**Model 3: Linear regression taking the RIBS scale (behaviors score) as dependent variable.**					
	**Unstandardized Beta**	**Standardized Beta**	**p-value**	**Confidence interval**	
				**Lower Bound**	**Upper Bound**
Attitudes toward Mental Illness (CAMI scale)	0.077	0.320	<0.001	0.067	0.087
Knowledge toward mental illness (MAKS scale)	0.115	0.193	<0.001	0.090	0.139
Familiarity with close people (yes vs no*)	0.577	0.119	<0.001	0.366	0.788
Familiarity with non-close person who have mental illness (yes vs no*)	-0.720	-0.087	<0.001	-1.082	-0.358
Variables entered: MAKS scale, CAMI scale, Familiarity with close people who have mental illness, Region, education level, familiarity with close people who have mental illness, familiarity with non-close person who have mental illness.					

*Reference group

CAMI: Community Attitudes toward Mental Illness; MAKS: Mental Health Knowledge Schedule; RIBS: reported and Intended Behavior Scale

## Discussion

To the best of our knowledge, this is the first study that assesses knowledge, attitude and behavior towards mentally ill patients in a Lebanese sample. The results obtained are in line with other studies [31, 32, 43, 44] showing a high prevalence of stigma toward mental illness in our sample. A study done by Abolfotouh et al. among the Saudi public had found that the majority of the sample (87.5%) reported lack of knowledge of mental illness, 66.5% had negative perception and 54.5% had negative attitudes to mental illness [45]. In a Moroccan study, most families (76%) reported having no knowledge about mental illness [46]. A study done by Coker et al. had found that 85.5% of the sample would not accept a psychotic person [31]. Other findings showed that only 38% of parents of children having mental illness in the United Arab Emirates would seek medical help [32]. Lebanese families are still denying the presence of mental illness and many individuals choose not to seek professional help out of fear of their communities’ reactions. There are still misconceptions and stigma associated with mental illness in the Lebanese population, similar to other Arab countries. Al-Krenawi et al. (2005) found that Arab patients with mental illness avoid the negative reactions of the public towards their illness by not disclosing their psychiatric symptoms to others [27].

In fact, the Lebanese society where religion plays an important role, pays special attention to beliefs about sin and causes and treatment of mental illness. Lower scores on public stigma against mental illness were associated with the belief that evil eye, magic and punishment from God might cause mental illness [33]. The theological view of mentally illness in the Arab culture considers that the illness is related to evil eye or a result from a sin or wrongdoing, with mental illness being a consequence of God punishment and a demonic possession [47]. The primary treatment is spiritual healing (exorcism to eradicate the demon or evil eye), miraculous healing through prayer and reading the holy book (Bible or Koran) [47]. In the Arab culture, the care of the ill person is the responsibility of the family [48]; Arab families tend to hold negative attitudes toward psychiatric services; it takes them months and even years to accept that the person with mental illness needs professional psychiatric care [28]. Nevertheless, the majority of Arab families still hold restrictive cultural and social beliefs that consider the mentally ill patients as a shame [49]. A study done by Dalky in 2012 found that Arab families perceived the experience of caring for a family member with a mental illness with fear, loss, embarrassment, and disgrace of family reputations [50]. Another study done by Kadri et al. among a sample of 100 Moroccan family members accompanying patients with schizophrenia showed that 86.7% of family members reported having hard/difficult lives and 72% reported psychological suffering and poor quality of life [46].

### Knowledge

Stigma has been associated with low knowledge about mental health disorders. There are two types of knowledge; the first refers to the people’s familiarity with various disorders such as depression and schizophrenia; this shows that they consider them as disorders so it is more likely for them to suggest help or care from a physician. The second refers to the high educational level being correlated to less prejudice and segregation towards mentally ill patients [51].

Moreover, our study has shown that people over 70 years old had less knowledge towards mental illness; our findings are consistent with those reported in an Indian study [52] but are in opposite to other studies that showed that older people have more knowledge because as they grow older, they are exposed to more experience and therefore more knowledge [53]. In addition, no correlation between age and knowledge was found according to a more recent study [54]. The association between age and knowledge towards mental illness is still controversial, with more in-depth research needed for a better understanding.

Knowing non-close people with mental illness was associated with higher knowledge according to the results of this study. These findings are in agreement with previous ones that showed that persons who are in contact with mentally-ill patients possess an adequate overall information about mental illness since they experience with those patients some of the signs, symptoms and treatments of the disease [53].

Finally, our results indicate that living in North Lebanon was associated with higher knowledge scores of mental illness, whereas living in Bekaa was associated with lower knowledge. In view of these results, awareness campaigns to increase knowledge of mental disorders should target the Bekaa region most importantly, but also the other regions since a lack of knowledge of mental illness apparently exists in all Lebanese governorates.

### Attitudes and behaviors

Attitudes and behaviors vary from positive and understanding to negative and repulsive. In our study, a positive correlation was established between the CAMI score and knowledge, so better attitudes are associated with more knowledge. Similar results were found in some studies [10, 11, 55–59] whereas others showed opposing results with no significant correlation between knowledge and attitude [60, 61]. One theory states that individuals with a higher level of knowledge have had the chance to get educated on the topic and are as a result more understanding and have better attitudes. Other studies showed that having higher level of knowledge of mental illness has forced people to be more distant from the mentally ill as they know their actual symptoms and behaviors [62, 63].

Gender is also a significant variable when it comes to stigma. Our results showed that females had a better attitude towards the mentally ill in the bivariate but not in the multivariable analysis. This is in concordance with previous studies as it was shown that females are more empathetic and open-minded [54, 64] and positive [21] showing less stigma [65]. No significant correlation was established between gender and behavior in our study; however, in a Swedish study, females showed fear and social distance as opposed to men [66]. Other studies demonstrated that women have less social restrictiveness and prejudice and misconceptions [67]. A study done by Elkington et al. (2012) demonstrated that a male’s stigma focuses on the diagnosis itself, while a female’s stigma depends on how the patients are perceived by society, so males have a realistic view while a female’s view is more subjective [68]. Additional research is necessary to clarify this association.

Familiarity and experience with people suffering from a mental illness have shown to be one the most crucial criteria that determine attitudes and behaviors [54]. Our questionnaire assessed familiarity with friends, close and distant people, in relation to attitudes and behaviors. A positive correlation was established in all cases. Previous studies have shown that having experienced mental illness with others leads to more positive attitudes [64]. In fact, those having mental illnesses in the family induced more benevolence and higher CAMI score than those without mentally ill family members. In other cases however, having experience with someone suffering from mental illnesses establishes negative attitudes [18]. According to Arkar and Eker, experienced individuals become realistic and sense the danger along with mental illnesses, which is why their attitude shifts negatively [69].

The results revealed that higher mean of knowledge, positive behaviors and positive attitudes scores toward mental illness was seen in persons living mostly in Beirut and Mount Lebanon. In these areas the majority of activities (economic, political, education and industrial) are concentrated [70]. The higher education institutes are located mainly in the center of the country along with the three main mental health hospitals in Lebanon [35]. Education may provide information about mental illness that might reduce the blame placed on mentally ill people and could change the stigmatizing attitudes toward them [71]. Also regular contact with mentally ill patients that occur in the overcrowded areas may reduce the social distance (discomfort fear and distrust emotions) toward mentally ill persons [71].

### Limitations and strengths

This study used a large sample, which included measures specifically targeted for the evaluation of stigma. It provides a first description of the level of stigma in the Lebanese population. Despite these strengths, there are some limitations associated with this study. The study is cross-sectional with a low level of evidence. The instruments used to assess the attitude, knowledge and behaviors toward mental illness had not yet been validated in the Lebanese context. An information bias could exist since the participants provide us with information using a self-reported questionnaire. The results could not be generalized to the entire population, since the group of 18–24 years, with university education, and unemployed was more represented. Our data could not be weighted to account for the multi-level sampling design in the absence of official numbers from the Lebanese government in terms of total population per governorate and subcategories by age and gender. Future research using alternative methods, such as stratified random sampling, might obtain a more complete view of level of stigma.

## Conclusion

In a country where mental health disorders and stigma prevail, it was important to assess the factors that contribute to public stigma. Knowledge, attitudes and behaviors were differently associated among different members of the Lebanese society. Our main finding was that more knowledge is associated with better behaviors and attitudes and therefore less stigma, which is why it is important to initiate awareness campaigns all over the country and especially in schools to prepare a more knowledgeable and open-minded society. As a result, people suffering from mental health disorders will not feel ashamed to seek the professional help that they need.

## Supporting information

S1 Table Questionnaire in Arabic. (DOCX)

S2 Table Questionnaire in English. (DOCX)

## References

[1] Definitions of Stigma and Discrimination. Disability Rights California. Available from: https://www.disabilityrightsca.org/system/files/file-attachments/CM0401.pdf.

[2] Corrigan PW, Watson AC. Understanding the impact of stigma on people with mental illness. World psychiatry. 2002;1(1):16 16946807 PMC1489832

[3] Corrigan PW, Shapiro JR. Measuring the impact of programs that challenge the public stigma of mental illness. Clinical Psychology Review. 2010;30(8):907–22. 10.1016/j.cpr.2010.06.004 20674114 PMC2952670

[4] Henderson C, Thornicroft G. Stigma and discrimination in mental illness: Time to Change. The Lancet. 2009;373(9679):1928–30. 10.1016/S0140-6736(09)61046-1 19501729

[5] Vaughan G, Hansen C. ‘Like Minds, Like Mine’: a New Zealand project to counter the stigma and discrimination associated with mental illness. Australasian Psychiatry. 2004;12(2):113–7. 10.1080/j.1039-8562.2004.02083.x 15715752

[6] Bratbo J, Vedelsby AK. ONE OF US: The national campaign for anti-stigma in Denmark. The Stigma of Mental Illness-End of the Story?: Springer; 2017 p. 317–38.

[7] Stuart H, Chen S-P, Christie R, Dobson K, Kirsh B, Knaak S, et al Opening minds in Canada: background and rationale. The Canadian Journal of Psychiatry. 2014;59(1_suppl):8–12. 10.1177/070674371405901s04 PMC4213755 25565705

[8] Drake RE, Bond GR, Essock SM. Implementing evidence-based practices for people with schizophrenia. Schizophrenia Bulletin. 2009;35(4):704–13. 10.1093/schbul/sbp041 19491315 PMC2696376

[9] Wei Y, McGrath PJ, Hayden J, Kutcher S. Mental health literacy measures evaluating knowledge, attitudes and help-seeking: a scoping review. BMC psychiatry. 2015;15(1):291. 26576680 10.1186/s12888-015-0681-9 PMC4650294

[10] Henderson C, Robinson E, Evans‐Lacko S, Corker E, Rebollo‐Mesa I, Rose D, et al Public knowledge, attitudes, social distance and reported contact regarding people with mental illness 2009–2015. Acta Psychiatrica Scandinavica. 2016;134:23–33. 10.1111/acps.12607 27426643 PMC6680221

[11] Hansson L, Stjernswärd S, Svensson B. Changes in attitudes, intended behaviour, and mental health literacy in the Swedish population 2009–2014: an evaluation of a national antistigma programme. Acta Psychiatrica Scandinavica. 2016;134:71–9. 10.1111/acps.12609 27426648

[12] Angermeyer MC, Holzinger A, Carta MG, Schomerus G. Biogenetic explanations and public acceptance of mental illness: systematic review of population studies. The British Journal of Psychiatry. 2011;199(5):367–72. 10.1192/bjp.bp.110.085563 22045945

[13] Parra F. Social tolerance of the mentally ill in the Mexican American community. International Journal of Social Psychiatry. 1985;31(1):37–45. 10.1177/002076408503100104 3972491

[14] Brockington IF, Hall P, Levings J, Murphy C. The community's tolerance of the mentally ill. The British Journal of Psychiatry. 1993;162(1):93–9. 8425146 10.1192/bjp.162.1.93

[15] Kobau R, Zack MM, Barile JP, Marshall C, Bornemann T, Otey EM, et al Attitudes toward mental illness: Results from the behavioral risk factor surveillance system. 2012.

[16] Vezzoli R, Archiati L, Buizza C, Pasqualetti P, Rossi G, Pioli R. Attitude towards psychiatric patients: a pilot study in a northern Italian town. European Psychiatry. 2001;16(8):451–8. 11777735 10.1016/s0924-9338(01)00606-x

[17] Phelan JC, Link BG. Fear of people with mental illnesses: The role of personal and impersonal contact and exposure to threat or harm. Journal of Health and Social Behavior. 2004;45(1):68–80. 10.1177/002214650404500105 15179908

[18] Lauber C, Nordt C, Falcato L, Rössler W. Factors influencing social distance toward people with mental illness. Community mental health journal. 2004;40(3):265–74. 15259631 10.1023/b:comh.0000026999.87728.2d

[19] Hackler AH. Contact and stigma toward mental illness: Measuring the effectiveness of two video interventions. 2011.

[20] Stuber JP, Rocha A, Christian A, Link BG. Conceptions of mental illness: Attitudes of mental health professionals and the general public. Psychiatric services. 2014;65(4):490–7. 10.1176/appi.ps.201300136 24430508 PMC4028068

[21] Angermeyer MC, Dietrich S. Public beliefs about and attitudes towards people with mental illness: a review of population studies. Acta Psychiatrica Scandinavica. 2006;113(3):163–79. 10.1111/j.1600-0447.2005.00699.x 16466402

[22] Link BG, Phelan JC, Bresnahan M, Stueve A, Pescosolido BA. Public conceptions of mental illness: labels, causes, dangerousness, and social distance. American journal of public health. 1999;89(9):1328–33. 10.2105/ajph.89.9.1328 10474548 PMC1508784

[23] Martin JK, Pescosolido BA, Tuch SA. Of fear and loathing: The role of'disturbing behavior,'labels, and causal attributions in shaping public attitudes toward people with mental illness. Journal of health and social behavior. 2000:208–23.

[24] Perry BL, Pescosolido BA, Martin JK, McLeod JD, Jensen PS. Comparison of public attributions, attitudes, and stigma in regard to depression among children and adults. Psychiatric Services. 2007;58(5):632–5. 10.1176/ps.2007.58.5.632 17463343

[25] Walker JS, Coleman D, Lee J, Squire PN, Friesen BJ. Children's stigmatization of childhood depression and ADHD: magnitude and demographic variation in a national sample. Journal of the American Academy of Child & Adolescent Psychiatry. 2008;47(8):912–20. 18596557 10.1097/CHI.0b013e318179961a

[26] Seeman N, Tang S, Brown AD, Ing A. World survey of mental illness stigma. Journal of affective disorders. 2016;190:115–21. 10.1016/j.jad.2015.10.011 26496017

[27] Al-Krenawi A. Mental health practice in Arab countries. Current Opinion in Psychiatry. 2005;18(5):560–4. 10.1097/01.yco.0000179498.46182.8b 16639119

[28] Dardas L, Simmons LA. The stigma of mental illness in A rab families: a concept analysis. Journal of psychiatric and mental health nursing. 2015;22(9):668–79. 10.1111/jpm.12237 26118332

[29] Aloud N, Rathur A. Factors affecting attitudes toward seeking and using formal mental health and psychological services among Arab Muslim populations. Journal of Muslim Mental Health. 2009;4(2):79–103.

[30] Youssef J, Deane FP. Factors influencing mental-health help-seeking in Arabic-speaking communities in Sydney, Australia. Mental Health, Religion & Culture. 2006;9(1):43–66.

[31] Coker EM. Selfhood and social distance: toward a cultural understanding of psychiatric stigma in Egypt. Social Science & Medicine. 2005;61(5):920–30. 15955396 10.1016/j.socscimed.2005.01.009

[32] Eapen V, Ghubash R. Help-seeking for mental health problems of children: preferences and attitudes in the United Arab Emirates. Psychological reports. 2004;94(2):663–7. 10.2466/pr0.94.2.663-667 15154199

[33] Rayan A, Fawaz M. Cultural misconceptions and public stigma against mental illness among Lebanese university students. Perspectives in psychiatric care. 2018;54(2):258–65. 10.1111/ppc.12232 28726343

[34] WHO-AIMS report on mental health system in Lebanon. World Health Organization and Ministry of Health. 2010. Retrieved from https://www.who.int/mental_health/who_aims_report_lebanon.pdf?ua=1. Accessed on January 20, 2019.

[35] Organization WH. WHO-AIMS report on mental health system in Lebanon. World Health Organization and Ministry of Health Retrieved from http://wwwwhoint/mental_health/who_aims_country_reports/en/indexhtml#L. 2010.

[36] Karam EG, Mneimneh ZN, Dimassi H, Fayyad JA, Karam AN, Nasser SC, et al Lifetime prevalence of mental disorders in Lebanon: first onset, treatment, and exposure to war. Plos medicine. 2008;5(4):e61 10.1371/journal.pmed.0050061 18384228 PMC2276523

[37] Aramouny C, Kerbage H, Richa N, Rouhana P, Richa S. Knowledge, Attitudes, and Beliefs of Catholic Clerics’ Regarding Mental Health in Lebanon. Journal of religion and health. 2019:1–20. 10.1007/s10943-018-00739-w 30661138

[38] Karam G. Vocational and Technical Education in Lebanon: Strategic Issues and Challenges. International Education Journal. 2006;7(3):259–72.

[39] Melki I, Beydoun H, Khogali M, Tamim H, Yunis K. Household crowding index: a correlate of socioeconomic status and inter-pregnancy spacing in an urban setting. Journal of Epidemiology & Community Health. 2004;58(6):476–80. 15143115 10.1136/jech.2003.012690 PMC1732777

[40] Evans-Lacko S, Little K, Meltzer H, Rose D, Rhydderch D, Henderson C, et al Development and psychometric properties of the mental health knowledge schedule. The Canadian Journal of Psychiatry. 2010;55(7):440–8. 10.1177/070674371005500707 20704771

[41] Taylor SM, Dear MJ. Scaling community attitudes toward the mentally ill. Schizophrenia bulletin. 1981;7(2):225 10.1093/schbul/7.2.225 7280561

[42] Evans-Lacko S, Rose D, Little K, Flach C, Rhydderch D, Henderson C, et al Development and psychometric properties of the reported and intended behaviour scale (RIBS): a stigma-related behaviour measure. Epidemiology and Psychiatric Sciences. 2011;20(3):263–71. 21922969 10.1017/s2045796011000308

[43] Shahrour TM, Rehmani RS. Testing psychiatric stigma in a general hospital in Saudi Arabia. Saudi medical journal. 2009;30(10):1336–9. 19838444

[44] Al-Krenawi A, Graham JR, Kandah J. Gendered utilization differences of mental health services in Jordan. Community mental health journal. 2000;36(5):501–11. 10994683 10.1023/a:1001963714338

[45] Abolfotouh MA, Almutairi AF, Almutairi Z, Salam M, Alhashem A, Adlan AA, et al Attitudes toward mental illness, mentally ill persons, and help-seeking among the Saudi public and sociodemographic correlates. Psychology research and behavior management. 2019;12:45 10.2147/PRBM.S191676 30679929 PMC6338115

[46] Kadri N, Manoudi F, Berrada S, Moussaoui D. Stigma impact on Moroccan families of patients with schizophrenia. The Canadian Journal of Psychiatry. 2004;49(9):625–9. 10.1177/070674370404900909 15503735

[47] Mathison LA. Mental health stigma in religious communities: Development of a quantitative measure. 2016.

[48] Dalky HF. Perception and coping with stigma of mental illness: Arab families’ perspectives. Issues in mental health nursing. 2012;33(7):486–91. 10.3109/01612840.2012.676720 22757601

[49] Dardas LA, Simmons LA. The stigma of mental illness in Arab families: a concept analysis. J Psychiatr Ment Health Nurs. 2015;22(9):668–79. 10.1111/jpm.12237 26118332

[50] Dalky HF. Perception and coping with stigma of mental illness: Arab families' perspectives. Issues Ment Health Nurs. 2012;33(7):486–91. 10.3109/01612840.2012.676720 . 22757601

[51] Pierce ML. Stigma and Knowledge: A Questionnaire and Literature Review: Cleveland State University; 2012.

[52] Das S, Phookun HR. Knowledge, attitude, perception and belief (KAPB) of patients’ relatives towards mental illness: A crosssectional study. Delhi Psychiatry Journal. 2014;17:48–59.

[53] Ahmed N, Baruah A. Awareness about mental illness among the family members of persons with mental illness in a selected District of Assam. Indian Journal of Social Psychiatry. 2017;33(2):171.

[54] Buizza C, Ghilardi A, Ferrari C. Beliefs and Prejudices Versus Knowledge and Awareness: How to Cope Stigma Against Mental Illness. A College Staff E-survey. Community Ment Health J. 2017;53(5):589–97. 10.1007/s10597-017-0116-9 . 28188389

[55] LORENZAMAGLIANO CDR, FIORILLO A, MALANGONE C, GUARNERP M, MARASCO C, MAJ M. Cause e conseguenze psicosociali della schizofrenia: le opinioni degli italiani. Epidemiologia e Psichiatria Sociale. 2003;12(3):187. 14610854 10.1017/s1121189x00002967

[56] Song L-Y, Chang L-Y, Shih C-Y, Lin C-Y, Yang M-J. Community attitudes towards the mentally ill: The results of a national survey of the Taiwanese population. International Journal of Social Psychiatry. 2005;51(2):162–76. 10.1177/0020764005056765 16048245

[57] Vibha P, Saddichha S, Kumar R. Attitudes of ward attendants towards mental illness: Comparisons and predictors. International Journal of Social Psychiatry. 2008;54(5):469–78. 10.1177/0020764008092190 18786908

[58] Barke A, Nyarko S, Klecha D. The stigma of mental illness in Southern Ghana: attitudes of the urban population and patients’ views. Social psychiatry and psychiatric epidemiology. 2011;46(11):1191–202. 10.1007/s00127-010-0290-3 20872212 PMC3192946

[59] Pattyn E, Verhaeghe M, Bracke P. Attitudes toward community mental health care: the contact paradox revisited. Community mental health journal. 2013;49(3):292–302. 10.1007/s10597-012-9564-4 23179045

[60] Schomerus G, Schwahn C, Holzinger A, Corrigan PW, Grabe H, Carta MG, et al Evolution of public attitudes about mental illness: a systematic review and meta‐analysis. Acta Psychiatrica Scandinavica. 2012;125(6):440–52. 10.1111/j.1600-0447.2012.01826.x 22242976

[61] Pescosolido BA, Martin JK, Long JS, Medina TR, Phelan JC, Link BG. “A disease like any other”? A decade of change in public reactions to schizophrenia, depression, and alcohol dependence. American Journal of Psychiatry. 2010;167(11):1321–30. 10.1176/appi.ajp.2010.09121743 20843872 PMC4429867

[62] Chong SA, Abdin E, Picco L, Pang S, Jeyagurunathan A, Vaingankar JA, et al Recognition of mental disorders among a multiracial population in Southeast Asia. BMC psychiatry. 2016;16(1):121. 27142577 10.1186/s12888-016-0837-2 PMC4855433

[63] Subramaniam M, Vaingankar J, Heng D, Kwok KW, Lim YW, Yap M, et al The Singapore Mental Health Study: an overview of the methodology. International Journal of Methods in Psychiatric Research. 2012;21(2):149–57. 10.1002/mpr.1351 22331628 PMC6878512

[64] Ewalds-Kvist B, Högberg T, Lützén K. Impact of gender and age on attitudes towards mental illness in Sweden. Nordic journal of psychiatry. 2013;67(5):360–8. 10.3109/08039488.2012.748827 23241018

[65] Subramaniam M, Abdin E, Picco L, Pang S, Shafie S, Vaingankar J, et al Stigma towards people with mental disorders and its components–a perspective from multi-ethnic Singapore. Epidemiology and psychiatric sciences. 2017;26(4):371–82. 10.1017/S2045796016000159 27018715 PMC5647661

[66] Ewalds-Kvist B, Hogberg T, Lutzen K. Impact of gender and age on attitudes towards mental illness in Sweden. Nord J Psychiatry. 2013;67(5):360–8. 10.3109/08039488.2012.748827 . 23241018

[67] Yuan Q, Abdin E, Picco L, Vaingankar JA, Shahwan S, Jeyagurunathan A, et al Attitudes to mental illness and its demographic correlates among general population in Singapore. PloS one. 2016;11(11):e0167297 10.1371/journal.pone.0167297 27893796 PMC5125689

[68] Elkington KS, Hackler D, McKinnon K, Borges C, Wright ER, Wainberg ML. Perceived mental illness stigma among youth in psychiatric outpatient treatment. Journal of Adolescent Research. 2012;27(2):290–317. 10.1177/0743558411409931 PMC8031474 33840885

[69] Arkar H, Eker D. Influence of having a hospitalized mentally ill member in the family on attitudes toward mental patients in Turkey. Social psychiatry and psychiatric epidemiology. 1992;27(3):151–5. 1621141 10.1007/BF00788762

[70] United Nations Office for the Coordination of Humanitarian Affairs. (UNOCHA). Lebanese Population—Central Administration of Statistics (CAS) year 2002 dataset. Available from: https://reliefweb.int/sites/reliefweb.int/files/resources/20140811-Beirut%20MountLebanonGovernorateProfile.pdf. [Last accessed Nov7, 2018].

[71] National Academies of Sciences E, Medicine. Ending discrimination against people with mental and substance use disorders: The evidence for stigma change: National Academies Press; 2016. 27631043

