# Supplementary material for: Knowledge, attitude and behaviors towards patients with mental illness: Results from a national Lebanese study
Source: PLoS One. 2019 Sep 16;14(9):e0222172. doi: 10.1371/journal.pone.0222172 (PMC6746362; doi:10.1371/journal.pone.0222172)
Supplement: S1 Table — (DOCX) [file pone.0222172.s001.docx]

**الموقف تجاه المرض النفسي أو العقلي.**

هذا إستطلاع للرأي في إيطار دراسة حول إدراك الناس ومدى فهمهم للمرض النفسي أو العقلي، ويتضمن أسئلة تتمحور حول موقف الأشخاص من الأمراض النفسية والعقلية، مدى معرفتهم بها وكيفية تعاملهم مع الأشخاص الذين يعانون منها.

على المستطلعين أن يحددوا الى أي مدى يوافقون أو يعارضون كل من الإفادات المطروحة.

**١**- المعلومات الشخصية

- **العمر**:

ما بين ١٨ و٢٩ سنة ما بين ٣٠ و٤٩ سنة ما بين ٥٠ و٦٩ سنة ٧٠ سنة وما فوق

- **الجنس**:

أنثى ذكر

- **مكان الإقامة**:

محافظة........................ قضاء............................ مكان السكن....................................

- **المستوى التعليمي**:

ابتدائي ثانوي جامعي مهني

- **المهنة:**

مهنة حرة موظف موظف في مؤسسة تابعة لقطاع الصحة عاطل عن العمل

متقاعد طالب غير قادر على العمل

- ما هو عدد الأشخاص المقيمين في المنزل؟ ..................................
- ما هو عدد غرف النوم الموجودة في المنزل؟ ................................

٢**- مقياسCAMI (community attitude towards mental illness)**

والعقلية النفسية الصحة في مشاكل من يعانون الذين الأشخاص

- **CAMI 1**لا يجب تسليمهم أية مسؤوليات.

1 أوافق بشدة 2 أوافق 3 لا أوافق ولا أعارض 4 أعارض 5 أعارض بشدة

- **CAMI 2** يجب عزلهم عن باقي المجتمع .

1 أوافق بشدة 2 أوافق 3 لا أوافق ولا أعارض 4 أعارض 5 أعارض بشدة

- **CAMI 3**يجب استبعادهم عن شغل منصب عام .

1 أوافق بشدة 2 أوافق 3 لا أوافق ولا أعارض 4 أعارض 5 أعارض بشدة

- **CAMI 4**لا يجب حرمانهم من حقوقه.

5 أوافق بشدة 4 أوافق 3 لا أوافق ولا أعارض 2 أعارض 1 أعارض بشدة

- **CAMI 5**يجب تشجيعهم على تحمّل مسؤوليّات الحياة الطبيعية.

5 أوافق بشدة 4 أوافق 3 لا أوافق ولا أعارض 2 أعارض 1 أعارض بشدة

- **CAMI 6**لا يجب استبعادهم عن محيطهم .

5 أوافق بشدة 4 أوافق 3 لا أوافق ولا أعارض 2 أعارض 1 أعارض بشدة

- **CAMI 7**أقلّ خطورة بكثير مما الناس تعتقد.

5 أوافق بشدة 4 أوافق 3 لا أوافق ولا أعارض 2 أعارض 1 أعارض بشدة

- **CAMI 8**لا أقبل أن أعيش بجوار أحد منهم.

1 أوافق بشدة 2 أوافق 3 لا أوافق ولا أعارض 4 أعارض 5 أعارض بشدة

- **CAMI 9**من الأفضل تجنّبهم.

1 أوافق بشدة 2 أوافق 3 لا أوافق ولا أعارض 4 أعارض 5 أعارض بشدة

- **CAMI 10**أفضل طريقة للتعامل معهم هي حجزهم وراء أبواب مقفلة .

1 أوافق بشدة 2 أوافق 3 لا أوافق ولا أعارض 4 أعارض 5 أعارض بشدة

- **CAMI 11**هناك شيء ما يختص بهم يسهّل تمييزهم عن الناس العاديين .

1 أوافق بشدة 2 أوافق 3 لا أوافق ولا أعارض 4 أعارض 5 أعارض بشدة

- **CAMI 12** كانوا محطّ سخرية لفترة طويلة من الزمن .

5 أوافق بشدة 4 أوافق 3 لا أوافق ولا أعارض 2 أعارض 1 أعارض بشدة

- **CAMI 13**بحاجة إلى مراقبة و ضبط تماماً كالأطفال .

1 أوافق بشدة 2 أوافق 3 لا أوافق ولا أعارض 4 أعارض 5 أعارض بشدة

- **CAMI 14**لا يجب معاملتهم على أنهم منبوذين من المجتمع.

5 أوافق بشدة 4 أوافق 3 لا أوافق ولا أعارض 2 أعارض 1 أعارض بشدة

- **CAMI 15**يجب عدم التشديد على حماية المجتمع منهم.

5 أوافق بشدة 4 أوافق 3 لا أوافق ولا أعارض 2 أعارض 1 أعارض بشدة

- **CAMI 16**يجب أن نكون متساهلين أكثر تجاههم.

5 أوافق بشدة 4 أوافق 3 لا أوافق ولا أعارض 2 أعارض 1 أعارض بشدة

- **CAMI 17**لا يستحقّون الشفقة.

1 أوافق بشدة 2 أوافق 3 لا أوافق ولا أعارض 4 أعارض 5 أعارض بشدة

- **CAMI 18**عالة على المجتمع.

1 أوافق بشدة 2 أوافق 3 لا أوافق ولا أعارض 4 أعارض 5 أعارض بشدة

- **CAMI 19**هناك خدمات كافية لهم.

1 أوافق بشدة 2 أوافق 3 لا أوافق ولا أعارض 4 أعارض 5 أعارض بشدة

- **CAMI 20**علينا تأمين أفضل رعاية لهم.

5 أوافق بشدة 4 أوافق 3 لا أوافق ولا أعارض 2 أعارض 1 أعارض بشدة

- **CAMI 21**يجب تخصيص مبلغ أكبر من قيمة الضرائب لعلاج المرض العقلي أو النفسي .

5 أوافق بشدة 4 أوافق 3 لا أوافق ولا أعارض 2 أعارض 1 أعارض بشدة

- **CAMI 22**تخصيص مبالغ كبيرة من المال لرعايتهم هو هدر لأموال الدولة .

1 أوافق بشدة 2 أوافق 3 لا أوافق ولا أعارض 4 أعارض 5 أعارض بشدة

- **CAMI 23**المرض العقلي أو النفسي لا يختلف عن باقي الأمراض.

5 أوافق بشدة 4 أوافق 3 لا أوافق ولا أعارض 2 أعارض 1 أعارض بشدة

- **CAMI 24**نظرياً أيّ إنسان ممكن أن يتعرّض لمرض نفسي أو عقلي .

5 أوافق بشدة أوافق 3 لا أوافق ولا أعارض 2 أعارض 1 أعارض بشدة

- **CAMI 25**أحد أهم الأسباب للمرض العقلي أو النفسي هو عدم القدرة على ضبط النفس أو فقدان الإرادة .

1 أوافق بشدة 2 أوافق 3 لا أوافق ولا أعارض 4 أعارض 5 أعارض بشدة

- **CAMI 26**عند ظهور أي عارض يدل على المرض العقلي أو النفسي يجب فوراً إحالة المريض إلى مستشفى للأمراض العقلية والنفسية.

1 أوافق بشدة 2 أوافق 3 لا أوافق ولا أعارض 4 أعارض 5 أعارض بشدة

- **CAMI 27**مستشفايات الأمراض العقلية والنفسية هي وسائل قديمة لمعالجة المرض النفسي أو العقلي .

5 أوافق بشدة 4 أوافق 3 لا أوافق ولا أعارض 2 أعارض 1 أعارض بشدة

- **CAMI 28**مستشفايات الأمراض النفسية و العقلية تبدو كسجون أكثر من كونها مكان للإهتمام بالمريض.

5 أوافق بشدة 4 أوافق 3 لا أوافق ولا أعارض 2 أعارض 1 أعارض بشدة

- **CAMI 29**إذا تزوّجت امرأة من رجل كان يعاني من مرض عقلي أو نفسي تعتبر غبيّة حتّى ولو أنّه قد تعافى كليّاً.

1 أوافق بشدة 2 أوافق 3 لا أوافق ولا أعارض 4 أعارض 5 أعارض بشدة

- **CAMI 30**أغلبيّة النساء اللّواتي كنّ مريضات في مستشفى للأمراض النفسية أو العقلية يمكن الإعتماد عليهنّ لرعاية الأطفال.

5 أوافق بشدة 4 أوافق 3 لا أوافق ولا أعارض 2 أعارض 1 أعارض بشدة

- **CAMI 31**أفضل علاج للعديد منهم هو أن يكونوا جزء من المجتمع الطبيعي .

5أوافق بشدة 4 أوافق 3 لا أوافق ولا أعارض 2 أعارض 1 أعارض بشدة

- **CAMI 32**ينبغي قدر الإمكان توفير خدمات الصّحة العقلية والنفسية.

5 أوافق بشدة 4 أوافق 3 لا أوافق ولا أعارض 2 أعارض 1 أعارض بشدة

- **CAMI 33**يجب أن يقبل السكان موقع مرافق الصحة النفسية والعقلية في جوارهم لتلبية إحتياجات المجتع المحلي.

5 أوافق بشدة 4 أوافق 3 لا أوافق ولا أعارض 2 أعارض 1 أعارض بشدة

- **CAMI 34**إن تحديد مرافق الخدمات الصّحة العقليّة أو النفسيّة في الأحياء السّكنية لا يعرّض السكان المحليّين للخطر.

5 أوافق بشدة 4 أوافق 3 لا أوافق ولا أعارض 2 أعارض 1 أعارض بشدة

- **CAMI 35**ليس لدى السكان ما يخشونه من الناس الذين يأتون إلى حيّهم للحصول على خدمات الصحّة العقلية أو النفسية.

5 أوافق بشدة 4 أوافق 3 لا أوافق ولا أعارض 2 أعارض 1 أعارض بشدة

- **CAMI 36**ينبغي إبقاء مرافق الصحة النفسية أو العقلية خارج الأحياء السكنية .

1 أوافق بشدة 2 أوافق 3 لا أوافق ولا أعارض 4 أعارض 5 أعارض بشدة

- **CAMI 37**لدى السكان سبب وجيه لمقاومة موقع خدمات الصّحة النفسية أو العقلية في حيّهم .

1 أوافق بشدة 2 أوافق 3 لا أوافق ولا أعارض 4 أعارض 5 أعارض بشدة

- **CAMI 38**عيش المرضى عقلياً أو نفسياً داخل اللأحياء السكنية قد يكون علاج جيد لهم ولكن المخاطر على السّكان كبيرة جداً.

1 أوافق بشدة 2 أوافق 3 لا أوافق ولا أعارض 4 أعارض 5 أعارض بشدة

- **CAMI 39**من المخيف التّفكير في الأشخاص الّذين يعانون من مشاكل عقلية أو نفسية الذين يعيشون في الأحياء السّكنية .

1 أوافق بشدة 2 أوافق 3 لا أوافق ولا أعارض 4 أعارض 5 أعارض بشدة

- **CAMI 40**وضع مرافق الصحة العقلية أو النّفسية في من منطقة سكنيّة يقلّل من مستوى الحي السّكني.

1 أوافق بشدة 2 أوافق 3 لا أوافق ولا أعارض 4 أعارض 5 أعارض بشدة

٣**- وصف الأمراض العقلية والنفسية**

DESأيّ من هذه الأشياء تشعر أنّها عادة ما تصف الشخص المريض عقليّاً أو نفسيّاً:

- **DES 1**شخص لديه نوبات خطيرة من الإكتئاب. كلا □ نعم □
- **DES 2**شخص غير قادر على اتّخاذ قرارات بسيطة في حياته الخاصّة. كلا □ نعم □
- **DES 3**شخص لديه "شخصيّات متعددة". كلا □ نعم □
- **DES 4**شخص ولد مع بعض الإضطرابات التي تؤثر على طريقة عمل الدماغ. كلا □ نعم □
- **DES 5**شخص لا يمكن أن يكون مسؤولاً عن أعماله. كلا □ نعم □
- **DES 6**شخص عنيف. كلا □ نعم □
- **DES 7**شخص يعاني من مرض "الفصام ". كلا □ نعم □
- **DES8**شخص يجب أن يبقى في مستشفى للأمراض العقليّة أو النفسيّة. كلا □ نعم □
- **DES 9**لا يوجد/لا أعلم/أخرى (حدّد)................................................

**٤- المعرفة الشخصية المتعلقة بالصحة النفسية والعقلية**

- **C1**معظم الناس الّذين يعانون من مشاكل في الصحة النفسية أو العقلية يريدون الحصول على عمل مدفوع الأجر.

6 أوافق بشدّة 5 أوافق 4 لا أوافق و لا أعارض 2 أعارض 1 أعارض بشدة 3 لا أعلم

- **C2**إذ كان أحد الأصدقاء لديه مشكلة في الصحة العقلية أو النفسية, أنت تعلم ما هي النصيحة المناسبة الّتي عليك تقديمها إليه لكي يحصل علي مساعدة مهنية.

6 أوافق بشدّة 5 أوافق 4 لا أوافق و لا أعارض 2 أعارض 1 أعارض بشدة 3 لا أعلم

- **C3**العلاج الطبي (الأدوية) يمكن أن يكون علاج فعال للأشخاص الّذين يعانون من مشاكل صحية, نفسية عقلية.

6 أوافق بشدّة 5 أوافق 4 لا أوافق و لا أعارض 2 أعارض 1 أعارض بشدة 3 لا أعلم

- **C4**العلاج النفسي (مثل العلاج الحديث أو الإستشارة الطبية) يمكن أن يكون علاجاً فعّالاً للأشخاص الّذين يعانون من مشاكل نفسية أو عقلية.

6 أوافق بشدّة 5 أوافق 4 لا أوافق و لا أعارض 2 أعارض 1 أعارض بشدة 3 لا أعلم

- **C5**يمكن للأشخاص الّذين يعانون من مشاكل نفسية أو عقلية أن يتعافوا كليّاً.

6 أوافق بشدّة 5 أوافق 4 لا أوافق و لا أعارض 2 أعارض 1 أعارض بشدة 3 لا أعلم

- **C6**معظم الأشخاص الّذين يعانون من مشاكل في الصحة النفسية أو العقلية يذهبون إلى أخصائي الرعاية الصحية للحصول على المساعدة.

6 أوافق بشدّة 5 أوافق 4 لا أوافق و لا أعارض 2 أعارض 1 أعارض بشدة 3 لا أعلم

- **C7**المشاكل في الصحة النفسية أو العقلية هي علامة ضعف.

1 أوافق بشدّة 2 أوافق 4 لا أوافق و لا أعارض 5 أعارض 6 أعارض بشدة 3 لا أعلم

- **C8**الإكتئاب دليل على ضعف شخصي أو عاطفي.

1 أوافق بشدّة 2 أوافق 4 لا أوافق و لا أعارض 5 أعارض 6 أعارض بشدة 3 لا أعلم

- **C9**المرض العقلي أو النفسي دليل على ضعف شخصي أو عاطفي.

1 أوافق بشدّة 2 أوافق 4 لا أوافق و لا أعارض 5 أعارض 6 أعارض بشدة 3 لا أعلم

- **C10**الجنون / الإكتئاب دليل على ضعف شخصي أو عاطفي.

1 أوافق بشدّة 2 أوافق 4 لا أوافق و لا أعارض 5 أعارض 6 أعارض بشدة 3 لا أعلم

- **C11**إلتماس العلاج لمشكلة في الصحة النفسية أو العقلية هي دليل ضعف.

1 أوافق بشدّة 2 أوافق 4 لا أوافق و لا أعارض 5 أعارض 6 أعارض بشدة 3 لا أعلم

- **C12**العلاج الطّبي (تناول الدواء) لمشكلة في الصحة النفسية أو العقلية هو دليل على ضعف شخصي أو عاطفي.

1 أوافق بشدّة 2 أوافق 4 لا أوافق و لا أعارض 5 أعارض 6 أعارض بشدة 3 لا أعلم

- **C13**العلاج النفسي لمشكلة في الصحة العقلية أو النفسية هي دليل على ضعف شخصي أو عاطفي.

1 أوافق بشدّة 2 أوافق 4 لا أوافق و لا أعارض 5 أعارض 6 أعارض بشدة 3 لا أعلم

يرجى أن تقول إلى مدى توافق أو لا توافق على أن كلّ من الحالات التالية هي نوع من الأمراض العقليّة أو النفسيّة :

- **C14الإكتئاب**:

5 أوافق بشدة 4 أوافق 3 لا أوافق ولا أعارض 2 أعارض 1 أعارض بشدة

- **C15الضغط النفسي**:

5 أوافق بشدة 4 أوافق 3 لا أوافق ولا أعارض 2 أعارض 1 أعارض بشدة

- **C16الفصام:**

5 أوافق بشدة 4 أوافق 3 لا أوافق ولا أعارض 2 أعارض 1 أعارض بشدة

- **C17اضطراب ثنائي القطب (المزاجيّة: اكتئاب/حالة ماسيّة):**

5 أوافق بشدة 4 أوافق 3 لا أوافق ولا أعارض 2 أعارض 1 أعارض بشدة

- **C18إدمان المخدرات:**

5 أوافق بشدة 4 أوافق 3 لا أوافق ولا أعارض 2 أعارض 1 أعارض بشدة

- **C19الفقدان أو الخسارة:**

5 أوافق بشدة 4 أوافق 3 لا أوافق ولا أعارض 2 أعارض 1 أعارض بشدة

٥**- التجربة الذاتية مع المرض النفسي والعقلي**

الأسئلة التالية تتعّلّق بخبراتك و آرائك بما يخص الأشخاص الّذين يعانون من مشاكل صحيةّ نفسيّة أو عقليّة؛ أعني بذلك الناس الّذين عوينوا من قبل مسؤولي الرعاية الصحيّة لمشكلة تتعلّق بالصّحة العقليّة أو النفسيّة.

- **E1**هل تعيش حالياً مع شخص يعاني من مشكلة تتعلّق بالصّحة العقلية أو النفسية، أم سبق لك أن عشت معه ؟

1 نعم 0 لا 2 لا أعلم

- **E2**هل تعمل حالياّ , أو سبق أن عملت مع شخص يعاني من مشكلة تتعلق بالصحة العقليّة أو النفسيّة ؟

1 نعم 0 لا 2 لا أعلم

- **E3**هل لديك حالياً , أو سبق أن كان لديك أحد من جيرانك يعاني من مشكلة في الصّحة العقلية أو النفسية؟

1 نعم 0 لا 2 لا أعلم

- **E4**هل لديك حالياً , أوكان لديك في وقت مضى , صديق مقرّب يعاني من مشكلة في الصّحة العقليّة؟

1 نعم 0 لا 2 لا أعلم

- **E5**في المستقبل، هل سوف تكون على استعداد للعيش مع شخص يعاني من مشكلة في الصحة العقلية أو النفسية؟

6 أوافق بشدّة 5 أوافق 4 لا أوافق و لا أعارض 2 أعارض 1 أعارض بشدة 3 لا أعلم

- **E6**في المستقبل، هل سوف تكون على استعداد للعمل مع شخص يعاني من مشكلة في الصحة العقلية أو النفسية. 6 أوافق بشدّة 5 أوافق 4 لا أوافق و لا أعارض 2 أعارض 1 أعارض بشدة 3 لا أعلم
- **E7**في المستقبل، هل سوف تكون على استعداد للعيش في مكان قريب لشخص يعاني من مشكلة في الصحة العقلية أو النفسية.

6 أوافق بشدّة 5 أوافق 4 لا أوافق و لا أعارض 2 أعارض 1 أعارض بشدة 3 لا أعلم

- **E8**في المستقبل، هل سوف تكون على استعداد لمواصلة العلاقة مع صديق قد عانى من مشكلة في الصحة العقلية أو النفسية .

6 أوافق بشدّة 5 أوافق 4 لا أوافق و لا أعارض 2 أعارض 1 أعارض بشدة 3 لا أعلم

- من هو الشخص الأقرب إليك الّذي لديه أو كان لديه مشكلة في الصحة العقلية أو النفسية ؟

**E9** الأسرة الصغيرة (الزوج / الطفل / الأخت / الأخ / الوالدين....). كلا □ نعم □

**E10** شريك (يعيش معك). كلا □ نعم □

**E11** شريك (لا يعيش معك). كلا □ نعم □

**E12** العائلة الكبيرة (عم / عمّة / ابن عم / الجدّ...). كلا □ نعم □

**E13** صديق. كلا □ نعم □

**E14** أحد معارفك. كلا □ نعم □

**E15** زميل عمل. كلا □ نعم □

**E16** أنت نفسك. كلا □ نعم □

**E17** غير ذلك (يرجى التحديد). كلا □ نعم □

**E18** لا أحد. كلا □ نعم □

- **E19**إذا شعرت أن لديك مشكلة في الصحة العقلية أو النفسية، ما مدى إحتمال أن تذهب إلى طبيبك العام للحصول على المساعدة؟

6 من المرجح جداً 5 على الأرجح 4لا رأي 2غير محتمل تماماً 1 من المستبعد 3 لا أعلم

- **E20**بشكل عام ما مدى شعورك بالراحة في التحدث مع صديق أو أحد أفراد عائلتك حول صحتك النفسية أو العقلية، على سبيل المثال بأن لديك مشكلة نفسية أو عقلية و كيف ذلك يؤثر عليك .

6 مريح جداً 5 مريح إلى حدّ ما 4لا مريحة ولا مزعج 2 مزعج إلى حدّ ما 1 مزعج للغاية 3 لا أعلم.

- **E21**بشكل عام ما مدى شعورك بالراحة في التحدث مع أحد أصحاب عملك الحالي أو المحتمل بشان صحتك النفسية أو العقلية و إخبارهم بأن لديك مشكلة نفسية أو عقلية و كيف يؤثرعليك ذلك ؟

6 مريح جداً 5 مريح إلى حدّ ما 4 لا مريحة ولا مزعج 2 مزعج إلى حدّ ما 1 مزعج للغاية 3لا أعلم.

- **E22**ما هي بنظرك نسبة الأشخاص في "لبنان" الّذين قد يعانون من مشاكل في الصحة النفسية أو العقلية في مرحلة ما في حياتهم ؟

1 ١ من أصل ١٠٠٠ 2 ١ من أصل١٠٠ 3 ١ من أصل ٥٠ 4 ١ من أصل ٤ 5 ١ من أصل ٣

**٦- وصمة العار المرتبطة بالصحة النفسية والعقلية وحملات التوعية**

- **S1** هل تعتقد أن الأشخاص الّذين يعانون من مرض عقلي أو نفسي يعانون من التمييز ويعتبروا أن لديهم “وصمة عار” في عصرنا الحالي ؟

1 نعم، يعانون من الكثير من وصمة العار والتمييز 2 نعم، يعانون من وصمة العار و التمييز 3 لا

- **S2**هل تعتقد أن وصمة العار و التمييز المرتبط بالصحة العقلية أو النفسية قد تغيّر في العام الماضي؟

1 نعم , تزايدت 3 نعم , انخفضت 2لا أعلم

- **S3**هل شاهدت أو سمعت أي إعلانات عن مشاكل الصحة العقلية أو النفسية خلال السنوات القليلة الماضية ؟

3 نعم , شاهدت أو سمعت هذه الإعلانات 1 لا , لم أرَ أو أسمع 2 لا أعلم

- **S4**إذا نعم , شاهدت أم سمعت هذه الإعلانات

كم مرّة من قبل هذه المقابلة , شاهدت أو سمعت أي إعلان عن مشاكل الصحة العقلية أو النفسية ؟

2 مرّة واحدة إلى مرّتين 3 ٣ إلى ٥ مرّات 4 ٦ مرّات أو أكثر 1 لا أعلم.
