# Supplementary material for: Knowledge, attitude and behaviors towards patients with mental illness: Results from a national Lebanese study
Source: PLoS One. 2019 Sep 16;14(9):e0222172. doi: 10.1371/journal.pone.0222172 (PMC6746362; doi:10.1371/journal.pone.0222172)
Supplement: S2 Table — (DOCX) [file pone.0222172.s002.docx]

**Attitude towards psychological and mental illness**

This is a survey in the framework of a study on people's understanding of psychological or mental illness. It includes questions about the attitude of people to psychological and mental illness, how they know about them and how they treat people who suffer from them.

The respondents should determine to what extent they agree or disagree with each of the statements made.

1. **Personal Information**
   - Age :
     1. Between 18 and 29 years
     2. Between 30 and 49 years
     3. Between 50 and 69 years
     4. Above 70 years
   - Gender
2. Male
3. Female
   - Address
4. Governorate:_________
5. District:_____________
6. Address:__________
   - Education level
7. Primary
8. Secondary
9. University
10. Technical
    - Career
11. Free lance
12. Employee
13. Healthcare employee
14. Unemployed
15. Retired
16. Student
17. Unable to work
    - What is the number of residents who live in your house?
    - How many rooms do you have in your house?
18. **Community attitude towards illness (CAMI)**

People who suffer from mental and psychological illnesses

- CAMI1: The mentally ill should not be given any responsibility.

1. Strongly disagree
2. Disagree
3. Neutral
4. Agree
5. Strongly agree

- CAMI2: The mentally ill should be isolated from the rest of the community

1. Strongly disagree
2. Disagree
3. Neutral
4. Agree
5. Strongly agree

- CAMI3: Anyone with a history of mental problems should be excluded from taking public office

1. Strongly disagree
2. Disagree
3. Neutral
4. Agree
5. Strongly agree

- CAMI4: The mentally ill should not be denied their individual rights.

1. Strongly disagree
2. Disagree
3. Neutral
4. Agree
5. Strongly agree

- CAMI5: Mental patients should be encouraged to assume the responsibilities of normal life.

1. Strongly disagree
2. Disagree
3. Neutral
4. Agree
5. Strongly agree

- CAMI6: The mentally ill should be isolated from the rest of the community.

1. Strongly disagree
2. Disagree
3. Neutral
4. Agree
5. Strongly agree

- CAMI7: The mentally ill are far less of a danger than most people suppose.

1. Strongly disagree
2. Disagree
3. Neutral
4. Agree
5. Strongly agree

- CAMI8 I would not want to live next door to someone who has been mentally ill.

1. Strongly disagree
2. Disagree
3. Neutral
4. Agree
5. Strongly agree

- CAMI9: It is best to avoid anyone who has mental problems.

1. Strongly disagree
2. Disagree
3. Neutral
4. Agree
5. Strongly agree

- CAMI10: The best way to handle the mentally ill is to keep them behind locked doors

1. Strongly disagree
2. Disagree
3. Neutral
4. Agree
5. Strongly agree

- CAMI11: There is something about the mentally ill that makes it easy to tell them from normal people.

1. Strongly disagree
2. Disagree
3. Neutral
4. Agree
5. Strongly agree

- CAMI12: The mentally ill have for too long been the subject of ridicule.

1. Strongly disagree
2. Disagree
3. Neutral
4. Agree
5. Strongly agree

- CAMI13: Mental patients need the same kind of control and discipline as a young child.

1. Strongly disagree
2. Disagree
3. Neutral
4. Agree
5. Strongly agree

- CAMI14: The mentally ill should not be treated as outcasts of society.

1. Strongly disagree
2. Disagree
3. Neutral
4. Agree
5. Strongly agree

- CAMI15: Less emphasis should be placed on protecting the public from the mentally ill.

1. Strongly disagree
2. Disagree
3. Neutral
4. Agree
5. Strongly agree

- CAMI16: we should be more easy going towards them

1. Strongly disagree
2. Disagree
3. Neutral
4. Agree
5. Strongly agree

- CAMI17: The mentally ill do not deserve our sympathy.

1. Strongly disagree
2. Disagree
3. Neutral
4. Agree
5. Strongly agree

- CAMI18: The mentally ill are a burden on society.

1. Strongly disagree
2. Disagree
3. Neutral
4. Agree
5. Strongly agree

- CAMI19: There are sufficient existing services for the mentally ill.

1. Strongly disagree
2. Disagree
3. Neutral
4. Agree
5. Strongly agree

- CAMI20: We have the responsibility to provide the best possible care for the mentally ill.

1. Strongly disagree
2. Disagree
3. Neutral
4. Agree
5. Strongly agree

- CAMI21: More tax money should be spent on the care and treatment of the mentally ill.

1. Strongly disagree
2. Disagree
3. Neutral
4. Agree
5. Strongly agree

- CAMI22: Increased spending on mental health services is a waste of tax dollars.

1. Strongly disagree
2. Disagree
3. Neutral
4. Agree
5. Strongly agree

- CAMI23: Mental illness is an illness like any other.

1. Strongly disagree
2. Disagree
3. Neutral
4. Agree
5. Strongly agree

- CAMI24: Virtually anyone can become mentally ill.

1. Strongly disagree
2. Disagree
3. Neutral
4. Agree
5. Strongly agree

- CAMI25: One of the main causes of mental illness is a lack of self-discipline and will power.

1. Strongly disagree
2. Disagree
3. Neutral
4. Agree
5. Strongly agree

- CAMI26: As soon as a person shows signs of mental disturbance, he should be hospitalized.

1. Strongly disagree
2. Disagree
3. Neutral
4. Agree
5. Strongly agree

- CAMI27: Mental hospitals are an outdated means of treating the mentally ill.

1. Strongly disagree
2. Disagree
3. Neutral
4. Agree
5. Strongly agree

- CAMI28: Our mental hospitals seem more like prisons than like places where the mentally ill can be cared for.

1. Strongly disagree
2. Disagree
3. Neutral
4. Agree
5. Strongly agree

- CAMI29: A woman would be foolish to marry a man who has suffered from mental illness, even though he seems fully recovered.

1. Strongly disagree
2. Disagree
3. Neutral
4. Agree
5. Strongly agree

- CAMI30: Most women who were once patients in a mental hospital can be trusted as baby sitters.

1. Strongly disagree
2. Disagree
3. Neutral
4. Agree
5. Strongly agree

- CAMI31: The best therapy for many mental patients is to be part of a normal community.

1. Strongly disagree
2. Disagree
3. Neutral
4. Agree
5. Strongly agree

- CAMI32: As far as possible mental health services should be provided through community based facilities.

1. Strongly disagree
2. Disagree
3. Neutral
4. Agree
5. Strongly agree

- CAMI33: Residents should accept the location of mental health facilities in their neighborhood to serve the needs of the local community.

1. Strongly disagree
2. Disagree
3. Neutral
4. Agree
5. Strongly agree

- CAMI34: Locating mental health services in residential neighborhoods does not endanger local residents

1. Strongly disagree
2. Disagree
3. Neutral
4. Agree
5. Strongly agree

- CAMI35: Residents have nothing to fear from people coming into their neighborhood to obtain mental health services.

1. Strongly disagree
2. Disagree
3. Neutral
4. Agree
5. Strongly agree

- CAMI36: Mental health facilities should be kept out of residential neighborhoods.

1. Strongly disagree
2. Disagree
3. Neutral
4. Agree
5. Strongly agree

- CAMI37: Local residents have good reason to resist the location of mental health services in their neighborhood.

1. Strongly disagree
2. Disagree
3. Neutral
4. Agree
5. Strongly agree

- CAMI38: Having mental patients living within residential neighborhoods might be good therapy, but the risks to residents are too great.

1. Strongly disagree
2. Disagree
3. Neutral
4. Agree
5. Strongly agree

- CAMI39: It is frightening to think of people with mental problems living in residential neighborhoods.

1. Strongly disagree
2. Disagree
3. Neutral
4. Agree
5. Strongly agree

- CAMI40: Locating mental health facilities in a residential area downgrades the neighborhood.

1. Strongly disagree
2. Disagree
3. Neutral
4. Agree
5. Strongly agree
6. **Description of mental and psychological diseases (dissociative experience scale DES)**

Which of the following is a habit that describes a mentally ill person?

- - DES1: Someone who has severe episodes of depression
  - DES2: someone who is not able to make simple decisions concerning his/her personal life
  - DES3: someone who has “multiple personalities”
  - DES4: a person born with disturbances that affect the functioning of the brain
  - DES5: someone who is unable to take responsibility over his/her duties
  - DES6: an aggressive person
  - DES7: someone who suffers from schizophrenia
  - DES8: someone who should stay in a psychiatric hospital
  - DES9:there aren’t any/ I don’t know/ other (precise)

1. **Personal knowledge about psychological and mental health**
   - C1: most people who suffer from mental and psychological problems want a paid job
2. Completely disagree
3. Disagree
4. I don’t know
5. Neither for nor against
6. Agree
7. Strongly agree

- C2: if you have a friend that you know suffers from a mental health issue, you know the proper advice to give to ensure that he/she gets the proper career guidance he/she needs
  - 1. Completely disagree
    2. Disagree
    3. I don’t know
    4. Neither for nor against
    5. Agree
    6. Strongly agree
- C3: medical treatment (medication) could be an effective treatment for mental and psychological illnesses

1. Completely disagree
2. Disagree
3. I don’t know
4. Neither for nor against
5. Agree
6. Strongly agree

- C4: psychological therapy could be an effective treatment for mental and psychological illnesses

1. Completely disagree
2. Disagree
3. I don’t know
4. Neither for nor against
5. Agree
6. Strongly agree

- C5: people who suffer from mental and psychological illnesses can be totally cured

1. Completely disagree
2. Disagree
3. I don’t know
4. Neither for nor against
5. Agree
6. Strongly agree

- C6: most people with psychological or mental health illnesses go to a healthcare professional for help

1. Completely disagree
2. Disagree
3. I don’t know
4. Neither for nor against
5. Agree
6. Strongly agree

- C7: mental and psychological illnesses are a sign of weakness

1. Completely disagree
2. Disagree
3. I don’t know
4. Neither for nor against
5. Agree
6. Strongly agree

- C8: depression is a sign of personal or emotional weakness

1. Completely disagree
2. Disagree
3. I don’t know
4. Neither for nor against
5. Agree
6. Strongly agree

- C9: mental and psychological illnesses are a sign of personal or emotional weakness

1. Completely disagree
2. Disagree
3. I don’t know
4. Neither for nor against
5. Agree
6. Strongly agree

- C10: madness/ depression is a sign of personal or emotional weakness

1. Completely disagree
2. Disagree
3. I don’t know
4. Neither for nor against
5. Agree
6. Strongly agree

- C11: seeking treatment for mental and psychological illnesses is a sign of weakness

1. Completely disagree
2. Disagree
3. I don’t know
4. Neither for nor against
5. Agree
6. Strongly agree

- C12: medical treatment (medications) for mental and psychological illnesses is a sign of personal and emotional weakness

1. Completely disagree
2. Disagree
3. I don’t know
4. Neither for nor against
5. Agree
6. Strongly agree

- C13:psychological therapy for mental and psychological illnesses is a sign of personal and emotional weakness

1. Completely disagree
2. Disagree
3. I don’t know
4. Neither for nor against
5. Agree
6. Strongly agree

Precise if you consider the following as mental and psychological health illnesses

- C14: depression

1. Completely disagree
2. Disagree
3. I don’t know
4. Neither for nor against
5. Agree
6. Strongly agree

- C15: psychological stress

1. Completely disagree
2. Disagree
3. I don’t know
4. Neither for nor against
5. Agree
6. Strongly agree

- C16: schizophrenia

1. Completely disagree
2. Disagree
3. I don’t know
4. Neither for nor against
5. Agree
6. Strongly agree

- C17: bipolar disorder (mood disorder: sad mood/ depression)

1. Completely disagree
2. Disagree
3. I don’t know
4. Neither for nor against
5. Agree
6. Strongly agree

- C18: drug addiction

1. Completely disagree
2. Disagree
3. I don’t know
4. Neither for nor against
5. Agree
6. Strongly agree

- C19: loss

1. Completely disagree
2. Disagree
3. I don’t know
4. Neither for nor against
5. Agree
6. Strongly agree
7. **Personal experience with mental and psychological illnesses**

The following questions concern your personal experiences and opinions concerning people who suffer from mental and psychological illnesses; I mean people who have been referred by health care officials due to a mental health problem.

E1: Do you or have you lived with someone who suffers from mental and psychological illnesses?

- 1. Yes
  2. No
  3. I don’t know
- E2: do you or have you worked with someone who suffers from mental and psychological illnesses?
  1. Yes
  2. No
  3. I don’t know
- E3: do you have or have you had any neighbors that suffer from mental and psychological illnesses?
  1. Yes
  2. No
  3. I don’t know
- E4: do you or have you had a close friend who suffers from mental and psychological illnesses?
  1. Yes
  2. No
  3. I don’t know
- E5: in the future, are you willing to live with someone who suffers from mental and psychological illnesses?
  1. I highly disagree
  2. I disagree
  3. I don’t know
  4. Neither with nor against
  5. I agree
  6. I highly agree
- E6: in the future, are you willing to work with someone who suffers from mental and psychological illnesses?
  1. I highly disagree
  2. I disagree
  3. I don’t know
  4. Neither with nor against
  5. I agree
  6. I highly agree
- E7: in the future, are you willing to live near with someone who suffers from mental and psychological illnesses?
  1. I highly disagree
  2. I disagree
  3. I don’t know
  4. Neither with nor against
  5. I agree
  6. I highly agree
- E8: in the future, are you willing to continue a friendship with someone who suffered from a mental health illness?
  1. I highly disagree
  2. I disagree
  3. I don’t know
  4. Neither with nor against
  5. I agree
  6. I highly agree

Who of the following suffers from mental or psychological illnesses?

- E9: immediate family (spouse/ child/ sister/ brother/ parents)
- E10: partner (live together)
- E11: partner (live separately)
- E12: extended family (uncle/ aunt/ cousin/ grandparent)
- E13: friend
- E14: One of your acquaintances
- E15: colleague
- E16: yourself
- E17: other (precise)
- E18: no one
- E19: if you feel that you have a mental health issue, how likely is it for to consult your general doctor for help?
  1. Not likely
  2. Impossible
  3. I don’t know
  4. No opinion
  5. Likely
  6. Very likely
- E20: how comfortable do you generally feel discussing your mental health issues with a friend or family member? For example, that you suffer from a mental health issue and how this affects you.
  1. Very uncomfortable
  2. Uncomfortable to a certain extent
  3. I don’t know
  4. Neither this nor the other
  5. Comfortable to a certain extent
  6. Very comfortable
- E21: how comfortable do you generally feel discussing your mental health issues with a colleague? For example, that you suffer from a mental health issue and how this affects you.
  1. Very uncomfortable
  2. Uncomfortable to a certain extent
  3. I don’t know
  4. Neither this nor the other
  5. Comfortable to a certain extent
  6. Very comfortable
- E22: what is the proportion of people in Lebanon who might suffer from a mental health issue during their life?
  1. 1 out of 1000
  2. 1 out of 100
  3. 1 out of 50
  4. 1 out of 4
  5. 1 out of 3

1. **Stigma against mental health illnesses and awareness campaigns**
   - S1: Do you think that people suffering from mental health issues are discriminate and stigmatized currently?
2. Yes, they suffer from a lot of stigma and discrimination
3. Yes, they suffer from stigma and discrimination
4. No

- S2: do you think that stigma and discrimination towards mental health issues have changed in the past year?

1. Yes, it has increased
2. I don’t know
3. Yes, it has decreased

- S3: have you seen or heard any advertisements concerning mental health issues in the past few years?

1. No, I have not seen or heard
2. I don’t know
3. Yes, I have seen or heard these advertisements

- S4: if yes, how many times have you seen or heard advertisements about mental health issues?

1. I don’t know
2. Once or twice
3. 3 to 5 times
4. 6 times or more
